# Moderating factors influence the relative age effect in Australian cricket

**DOI:** 10.7717/peerj.6867

**Published:** 2019-05-17

**Authors:** Jonathan D. Connor, Ian Renshaw, Kenji Doma

**Affiliations:** 1 College of Healthcare Sciences, Sport and Exercise Science, James Cook University, Townsville, Queensland, Australia; 2 Department of Sport Sciences and Sport Medicine, National Cricket Centre, Brisbane, Queensland, Australia; 3 School of Exercise and Nutrition Sciences, Queensland University of Technology, Brisbane, QLD, Australia

**Keywords:** Handedness, Talent selection, Relative age, Talent development, Gender, Fighting hypothesis, Maturation

## Abstract

**Background:**

The relative age effect is a commonly occurring phenomenon whereby there is a tendency for relatively older players to be over-represented during high level competitions. This effect is often seen to diminish as player’s age, however, there has been far less investigation on other potential moderating factors.

**Method:**

This study investigated the impact of the relative age effect, and potential moderating factors, within the talent selection process of Australian cricket. Relative age distribution of 2,415 male and female junior and senior state level cricket players, who played in the Junior National Championships or State competition (senior level) between 2011 and 2015, were analysed.

**Results:**

Players born in the first quartile of the cricket season were significantly over-represented in both male Under-15, Under-17, Under-19 and female Under-15 and Under-18 levels. However, there was no significant difference at the senior state level for either male or female cricketers. Further investigation of the relative age effect in the junior talent pathway revealed that male all-rounders, batters and pace bowlers, and female all-rounders and batters, born in first quartile were over-represented. Right-handed batters and bowlers were also influenced by the relative age effect at all Junior National levels, while left-handed batters and bowlers were only influenced at the Under-15 and Under-17 levels. These results highlight the impact relative age has on junior cricket talent pathways, including sex, age, handedness and primary skills. Only state level, and left-handedness at the Under-19 level, were unaffected by relative age.

**Discussion:**

The findings of this study highlight the influence of relative age effects for both male and female junior cricket players. Interestingly, there may be an advantage to being left-handed that is more prevalent at the older (male Under-19; female Under-18) age levels.

## Introduction

Analysing junior representative level competitions that are responsible for the selection and development of young talented athletes, have been ideal settings to examine relative age effects (RAE). Sports such as soccer (Gil et al., 2014; Van den Honert, 2012; Helsen, Van Winckel & Williams, 2005), tennis (Ulbricht et al., 2015), Australian Football (Cripps et al., 2015), athletics (Brazo-Sayavera et al., 2017) and rugby league (Till et al., 2010) have all reported (Coutts, Kempton & Vaeyens, 2014) instances of the RAE influencing selection at a representative level. Over-representation of relatively older players has been attributed to the likelihood of these players having attained a greater degree of physical maturation compared to their younger counter-parts (maturation-selection hypothesis; Brazo-Sayavera et al., 2017). However, these advantages are temporary, as both the prevalence of the RAE, and the disparity in physical maturity between players, decreases as players age (Musch & Grondin, 2001). For example, Woods, Robertson & Gastin (2015) reported that an Under-18 years AFL academy squad did not in fact over-select relatively older players, nor did players born early in the sporting year possess superior anthropometric or physical abilities. It has been argued that relative age may influence sports selection when physical maturity provides a significant advantage to a performer (Romann & Fuchslocher, 2014).

Alongside age level, sex and handedness have been identified as potential moderating factors of relative age (Schorer et al., 2009). While these factors have been scarcely explored, preliminary evidence suggests that RAE may be present only in certain sports and dependent on sex. For example, Nakata & Sakamoto (2012) compared multiple sports at a representative level between sex, highlighting an over-representation of relatively older male players in baseball, soccer, and track and field, while females exhibited the same phenomenon only in volleyball. Brazo-Sayavera and colleagues (2017) reported data from athletics where females were not affected by RAE, unlike their male counterparts of the same age. Two explanations have been proposed to explain these findings. Musch & Grondin (2001) highlighted that competition amongst selection is a necessary condition for RAE to occur. They argued that if the playing population pool within a particular sport is relatively small, it is unlikely that relative age will impact selection as there are ample opportunities for almost all players to be selected. Rather, the prevalence of RAE is greater in sports with a large playing population and a lower number of selection opportunities (Helsen, Starkes & Van Winckel, 1998). A secondary explanation, building upon the maturation hypothesis, is that females generally mature much earlier during their adolescence than males (Tanner & Whitehouse, 1976) and the physical development and morphological changes associated with puberty would suggestibly influence females at a younger age. Further research examining RAE in female athletes across multiple age groups is required.

While physical maturity is considered a critical factor of RAE (Musch & Grondin, 2001), sports involving a multitude of skills may exhibit similar phenomena. Cricket is an ideal example of a multi-skilled sport, whereby players are selected based on their dominant skill attributes. This includes batting, all-rounders (skilled at batting and one form of bowling), wicketkeeping, spin bowling or pace bowling. The importance of physical characteristics such as size and strength has yet to be established for cricket batting. In contrast, fast bowling is more reliant on superior anthropometric and physical characteristics in both male (Bartlett et al., 1996) and female (Stuelcken, Pyne & Sinclair, 2007) bowlers. Alternatively, skilled performance in spin bowling has been associated with greater coordinative control (e.g., pelvic rotation; Chin et al., 2009) rather than any specific morphologies. Finally, wicketkeeping, which is primarily a catching task, is unlikely to be advantaged by greater physical maturity and may be less likely influenced by RAE. Collectively, RAE may affect talent selection in cricket not only due to biological maturation, but by whether maturation can affect the overall performance of the skill.

Finally, dominant handedness has been described as another potential moderating factor, with left-handedness offering unique advantages during the early stages of talent development (Wattie, Schorer & Baker, 2015). For example, left-handed batters in cricket have previously been reported to have a greater scoring average during a national competition than their right-handed counterparts (Brooks et al., 2004). Regarding relative age, left-handers in some sports have been shown to be unaffected, unlike their right-handed counter-parts of the same age (Loffing, Schorer & Cobley, 2010). While a neurological explanation has been proposed (see Geschwind & Galaburda, 1987), the more prevalent theory is an innate strategic advantage; coined the ‘fighting hypothesis’ (Raymond et al., 1996; Wood & Aggleton, 1989). This theory highlights an inherent advantage possessed by players with unorthodox actions (such as left-handers), due to the lack of exposure players have competing against those types of performers (also see Goulet, Bard & Fleury, 1989). It is currently unclear whether dominant handedness weakens the prevalence of RAE during the adolescent years of talent selection.

The FTEM (Foundations, Talent, Elite, and Mastery) framework is a useful model in which to examine how RAE may impact players at the Talent level, through to the Elite level (Gulbin et al., 2013). For example, the early stages of the Talent level centres on demonstrating potential, and verification of that potential. However, this process may be biased towards relatively older players possessing an innate, albeit temporary, physical advantage over their peers.

The purpose of this study was to examine common moderating factors of the RAE in Australian cricket, including age, sex, cricket-specific skills and handedness. It is hypothesised that the RAE will be most prevalent in younger age groups for both males and females, gradually reducing until (adult state level). That is, relatively older males will be over-represented at the Under-15 (U15), Under-17 (U17) and Under-19 (U19) representative levels with similar findings observed for older females in their age group competitions at U15 age group. Dominant skills that are most impacted by early physical maturational factors, such as batting, fast bowling and fast bowling all-rounders are hypothesised to be over-represented by relatively older players. Finally, left-handers are hypothesised to be less susceptible to the effect of relative age. Additionally, those relatively older players will not be over-represented at the highest age group competition for both males (U19) and females (U18) for both batting and bowling.

## Methods

Playing data for all male U15, U17 and U19 and female U15 and U18 junior cricketers who competed in the Annual National Junior championships in Australia between 2011 and 2015 was obtained from an open source online database (nationalchamps.com.au), while access to non-identifiable data (i.e., birth date, dominant skill and handedness) was provided by the national organisation (Cricket Australia). Taking into consideration the cut-off date in age group competitions in Australian cricket of August 31st, player’s relative age was determined by coding player birth-dates into quartiles (Q1: September–November; Q2: December–February; Q3: March–May; Q4: June–August). While previous research on relative ages have often compared birth distribution of their group to an ‘assumption of equal distribution’ (Loffing, Schorer & Cobley, 2010), this study compared skilled cricketers birth distribution to the birthdates of all Australian club cricketers who played in 2015/2016 (Table 1). All players were informed of the activities of the program and voluntarily agreed to participate. This research received institutional approval from James Cook University (H6267) and was conducted in accordance with the National Statement on Ethical Conduct in Human Research (2007).

**Table 1:** Birth quartile distribution of Australian junior and senior club cricketers who played in the 2015/2016 season.

	Q1	Q2	Q3	Q4
Australian cricketers	50,063 (25.13%)	55,630 (27.93%)	46,808 (23.50%)	46,682 (23.44%)

### Statistical analysis

All data are presented as descriptive statistics, including the number of cricket players and their relative percentages. Chi-square analyses were utilised to compare the number of cricket players across different birth quartile distributions (Q1, Q2, Q3 and Q4) who played in the Australian National Cricket Championships between the 2012 and 2015 seasons inclusive. Within each player’s age group for male (U15, U17, U19 and state level) and female (U15, U18 and state level) cricket players, they were then further separated into their cricket-specific skill (all-rounders, batters, pace bowlers, spin bowlers and wicketkeepers) and subsequent dominant handedness (left or right) within batting or bowling skill. This was compared to the birth statistics (*n* = 199,183) of Australian cricketers who played in the 2014–2015 competition. Statistical analyses were conducted using SPSS version 22.0 software, while the alpha level was set at 0.05.

## Results

### Age and sex

**Figure 1:**
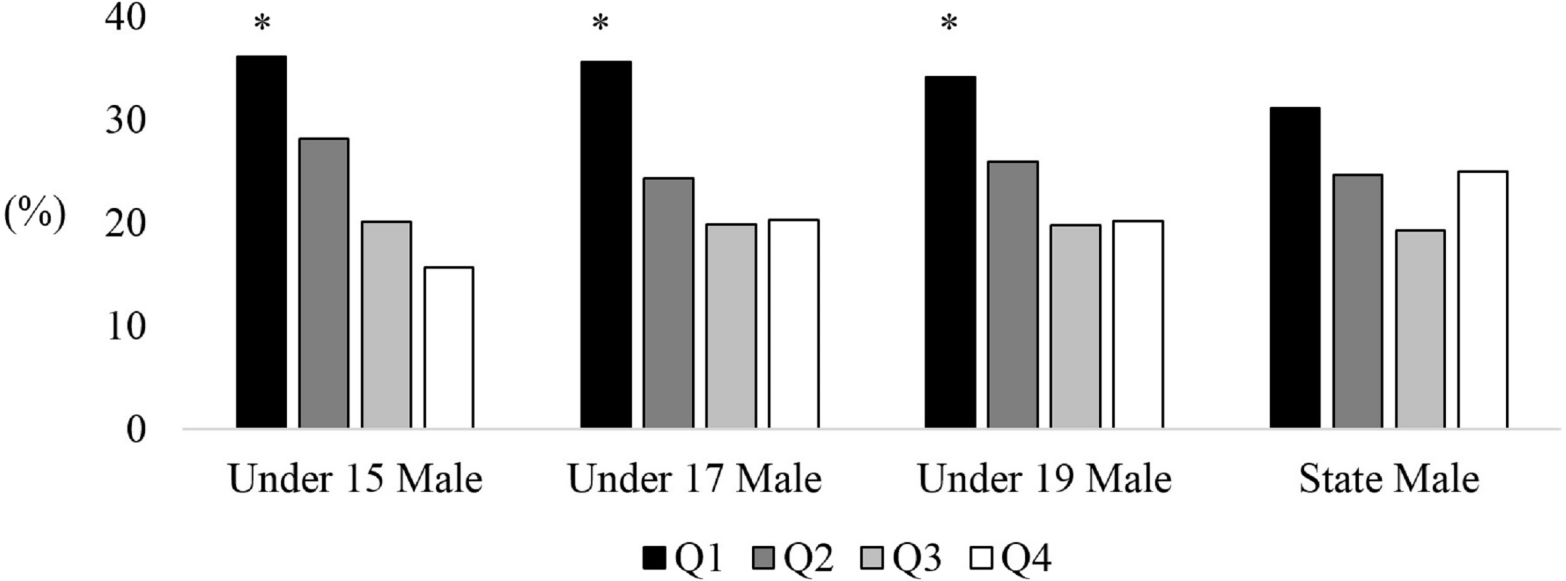
Birth quartiles of U15, U17 and U19 junior state level male cricketers. *Statistically significant finding (*p* < 0.05).

**Figure 2:**
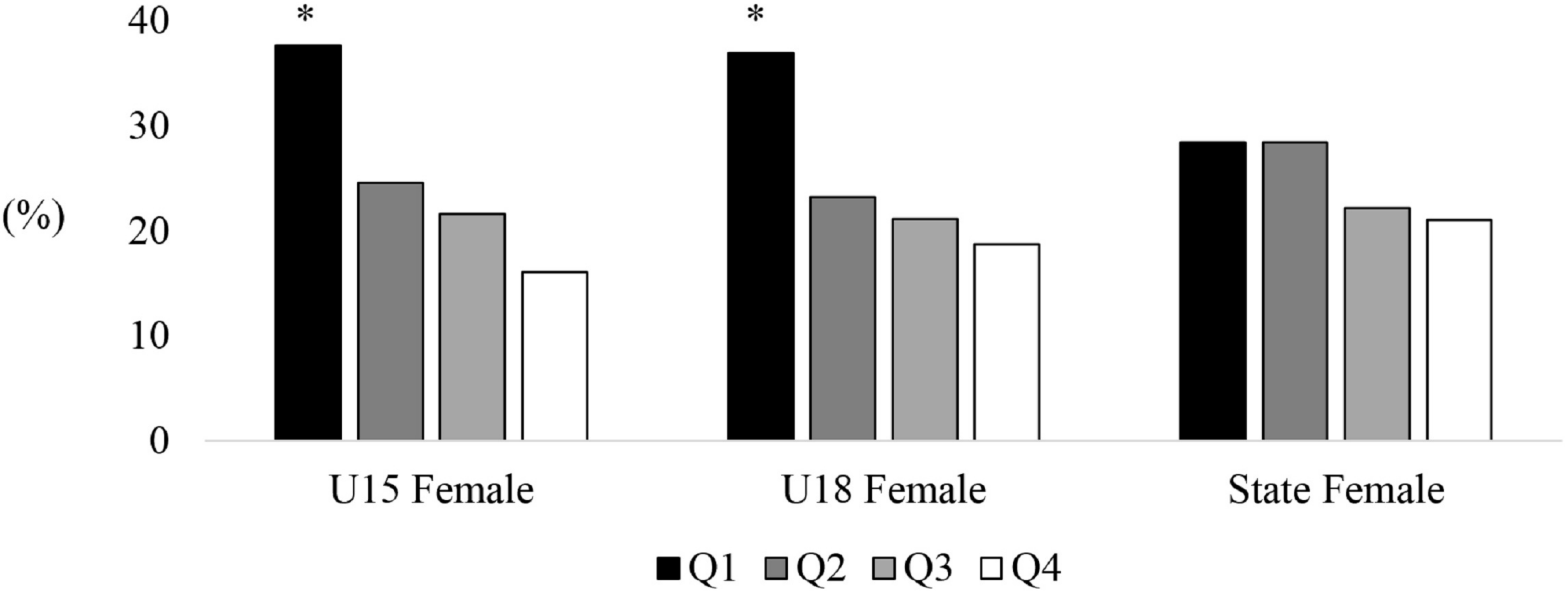
Birth quartiles of U15 and U18 junior state level female cricketers. *Statistically significant finding (*p* < 0.05).

A main effect of birth distribution was found for U15 (*X*^2^ = 26.13, *p* < 0.05; Fig. 1), U17 (*X*^2^ = 26.50, *p* < 0.05) and U19 (*X*^2^ = 20.00, *p* < 0.05) male cricketers participating in the Annual National Championships, however, no significant effect was found for state level players (*X*^2^ = 6.61, *p* = 0.08). There was an over-representation of players born in the first quartile of the year across U15 (36.1%), U17 (35.5%) and U19 (34.2%) age levels, while the third (U15, 20.1%; U17, 19.9%; U19, 19.7%) and fourth birth quartile (U15, 15.7%; U17, 20.3%; U19, 20.2%) were consistently lower than the expected value (Table 1). A main effect was also found for U15 (*X*^2^ = 34.27, *p* < 0.05; Fig. 2) and U18 (*X*^2^ = 28.55, *p* < 0.05) female cricketers in the National Championships, however, no effect was found for female state level players (*X*^2^ = 1.34, *p* = 0.72). Similar to male cricketers, both U15 and U18 female cricketers had an over-represented percentage of players born in the first quartile (37.7% and 37.0%), while the fourth quartile (16.0% and 18.7%) were the lowest represented.

### Cricket-specific skills

A main effect was reported for all cricket-specific skills across the male and female player pathway at one or more age levels. However, no effect was reported for any skill at state level for either male or female players. Birth distributions for male all-rounders were significant at the U15 and U17 level, however, no difference was found at the U19 or State level. The percentage of players born in the first quartile was the highest among birth distributions for all age levels, while the third and fourth quartile were all lower than the expected value (see Tables 1 and 2). A main effect was also found for batting in the U15 and U19 age group levels, and all age level groups for bowlers, with the first quartile being over-represented and the last quartile under-represented. No effects were reported for spin bowlers or wicketkeepers across any age level.

**Table 2:** Positional Breakdown of Under-15, Under-17 and Under-19 junior state level male cricketers over 4 years and their birth quartile.

Birth Quartiles											
	*n*	Q1		Q2		Q3		Q4		*X*^2^	*p*-value
**All-rounder**							
State Under-15^*^	*71*	32	(45.1%)	16	(22.5%)	10	(14.1%)	13	(18.3%)	16.27	<0.05
State Under-17^*^	*79*	34	(43.0)%	18	(22.8%)	13	(16.5%)	14	(17.7%)	14.41	<0.05
State Under-19	*72*	24	(33.3%)	19	(26.4%)	13	(18.1%)	16	(22.2%)	3.66	0.30
State Level	*39*	15	(38.5%)	7	(18.0%)	8	(20.5%)	9	(23.1%)	3.97	0.26
**Batter**											
State Under-15^*^	*132*	46	(34.9%)	37	(28.0%)	27	(20.5%)	22	(16.7%)	10.36	<0.05
State Under-17	*180*	60	(33.3%)	49	(27.2%)	37	(20.6%)	34	(18.9%)	9.47	0.07
State Under-19^*^	*182*	62	(34.1%)	52	(28.6%)	39	(21.4%)	29	(15.9%)	13.82	<0.05
State Level	*96*	29	(30.2%)	24	(25.0%)	19	(19.8%)	24	(25.0%)	2.08	0.56
**Pace Bowler**											
State Under-15^*^	*74*	26	(35.1%)	24	(32.4%)	17	(23.0%)	7	(9.5%)	11.95	<0.05
State Under-17^*^	*120*	49	(40.8%)	28	(23.3%)	24	(20.0%)	19	(15.8%)	17.40	<0.05
State Under-19^*^	*113*	47	(41.6%)	26	(23.0%)	21	(18.6%)	19	(16.8%)	17.51	<0.05
State Level	*73*	19	(26.0%)	22	(30.1%)	14	(19.2%)	18	(24.7%)	1.80	0.62
**Spin Bowler**											
State Under-15	*24*	4	(16.7%)	8	(33.3%)	5	(20.8%)	7	(29.2%)	1.67	0.64
State Under-17	*49*	13	(26.5%)	11	(22.5%)	10	(20.4%)	15	(30.6%)	1.20	0.75
State Under-19	*54*	13	(24.1%)	16	(29.6%)	11	(20.4%)	14	(25.9%)	0.96	0.81
State Level	*24*	8	(33.3%)	5	(20.8%)	4	(16.7%)	7	(29.2%)	1.67	0.64
**Wicketkeeper**											
State Under-15	*20*	7	(35.0%)	3	(15.0%)	8	(40.0%)	2	(10.0%)	5.20	0.16
State Under-17	*24*	5	(20.8%)	3	(12.5%)	6	(25.0%)	10	(41.7%)	4.33	0.23
State Under-19	*30*	8	(26.7%)	4	(13.3%)	5	(16.7%)	13	(43.3%)	6.53	0.09
State Level	*12*	5	(41.7%)	2	(16.7%)	2	(16.7%)	3	(25.0%)	2.00	0.57

**Notes.**

* Statistically significant finding (*P* < 0.05).

A main effect was found for female all-rounders at the U15 and U18 levels, with the first birth quartile over-represented across both age levels. No significant difference was found for female pace bowlers at the U15 or U18 age level. Female U15 spin bowlers were over-represented in the second quartile however no difference was found at the U18 level. Finally, a main effect was found for female U18 wicketkeepers, however, not at the U15 level.

### Handedness for Batters

**Figure 3:**
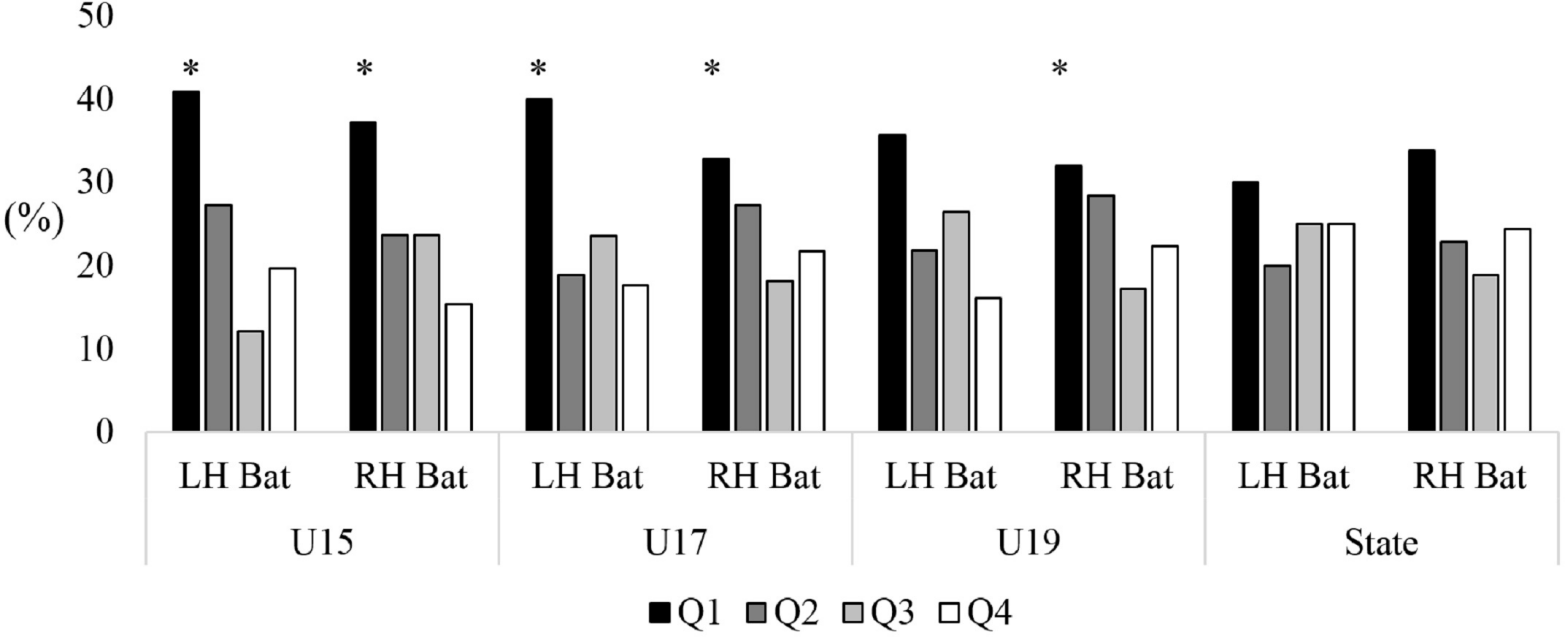
Comparison of birth quartiles and handedness (LH, left hand; RH, right hand) for primary skilled batters, all-rounders and wicketkeepers for junior representative male cricketers. *Statistically significant finding (*p* < 0.05).

Analysis of batter’s handedness found a significant over-representation of those born in the first quartile for male left-handed (*X*^2^ = 11.94, *p* < 0.05 [Q1: 40.9%; Q2: 27.3%; Q3: 12.1%; Q4: 19.7%]) and right-handed U15 batters (*X*^2^ = 15.23, *p* < 0.05 [Q1: 38.29%; Q2: 23.7%; Q3: 23.7%; Q4: 15.4%]), left-handed (*X*^2^ = 10.85, *p* < 0.05 [Q1: 40.0%; Q2: 18.8%; Q3: 23.5%; Q4: 17.6%]) and right-handed (*X*^2^ = 9.80, *p* < 0.05 [Q1: 32.8%; Q2: 27.3%; Q3: 18.2%; Q4: 21.7%]) U17 batters, and right-handed (*X*^2^ = 10.84, *p* < 0.05 [Q1: 32.0%; Q2: 28.4%; Q3: 17.3%; Q4: 22.3%]) U19 batters. No difference was evident for left-handed (*X*^2^ = 6.13, *p* = 0.06 [Q1: 35.6%; Q2: 21.8%; Q3: 26.4%; Q4: 16.1%]) U19 level batters, or left (*X*^2^ = 0.40, *p* = 0.94 [Q1: 30.0%; Q2: 20.0%; Q3: 25.0%; Q4: 25.0%]) or right-hand (*X*^2^ = 6.13, *p* = 0.11 [Q1: 33.9%; Q2: 22.8%; Q3: 18.9%; Q4: 24.4%]) State level batters (see Fig. 3).

**Figure 4:**
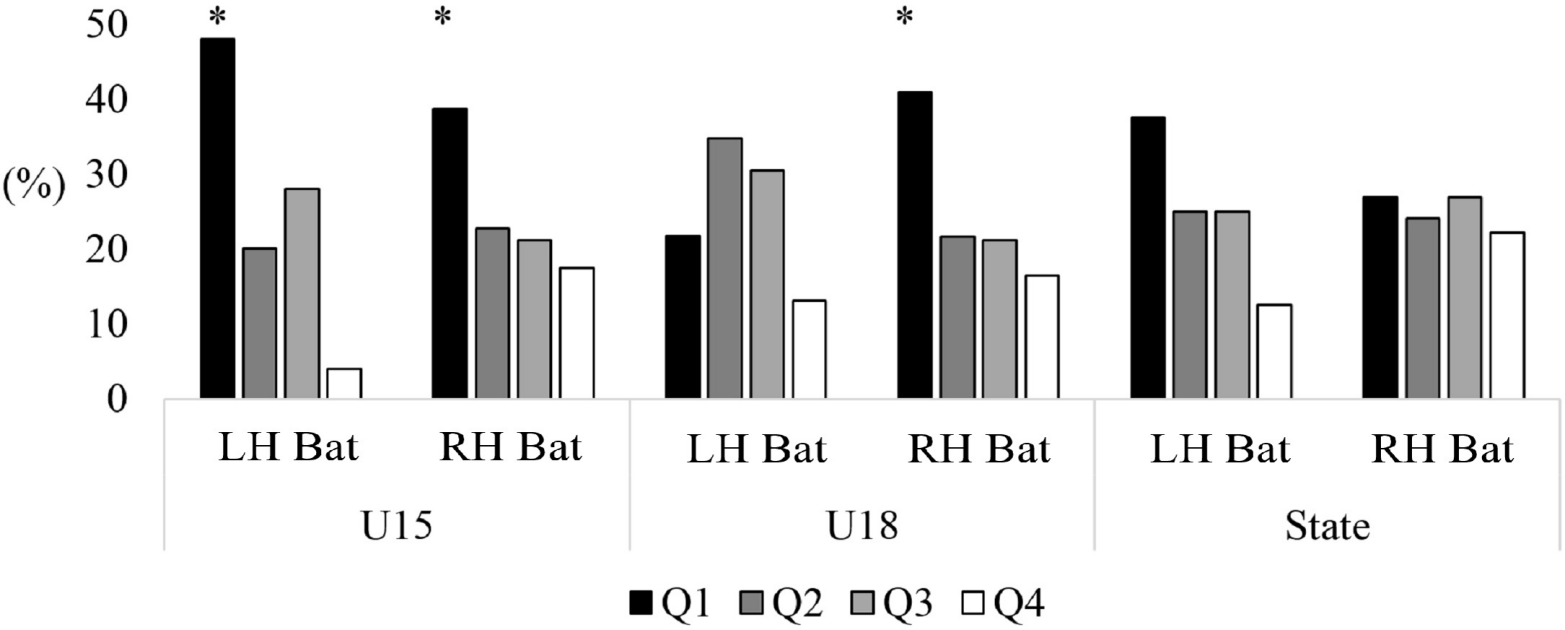
Comparison of birth quartiles and handedness for primary skilled batters, all-rounders and wicketkeepers for junior representative female cricketers. *Statistically significant finding (*P* < 0.05).

For female batters, significant effects were found for left-handed (*X*^2^ = 10.04, *p* < 0.05 [Q1: 48.0%; Q2: 20.0%; Q3: 28.0%; Q4: 4.0%]) and right-handed (*X*^2^ = 25.77, *p* < 0.05 [Q1: 38.6%; Q2: 22.8%; Q3: 21.1%; Q4: 17.5%]) U15 batters, and right-handed (*X*^2^ = 10.85, *p* < 0.05 [Q1: 40.9%; Q2: 21.6%; Q3: 21.1%; Q4: 16.4%]) U18 batters. In all groups, Q1 was the most represented in each group, while Q4 was the least represented. No difference was reported for left-handed (*X*^2^ = 2.57, *p* = 0.46 [Q1: 21.7%; Q2: 34.8%; Q3: 30.4%; Q4: 13.0%]) U18 level batters or left (*X*^2^ = 1.00, *p* = 0.80 [Q1: 37.5%; Q2: 25.0%; Q3: 25.0%; Q4: 12.5%]) or right-handed (*X*^2^ = 0.69, *p* = 0.88 [Q1: 26.9%; Q2: 24.0%; Q3: 26.9%; Q4: 22.1%]) State level (see Fig. 4).

### Handedness for Bowlers

**Figure 5:**
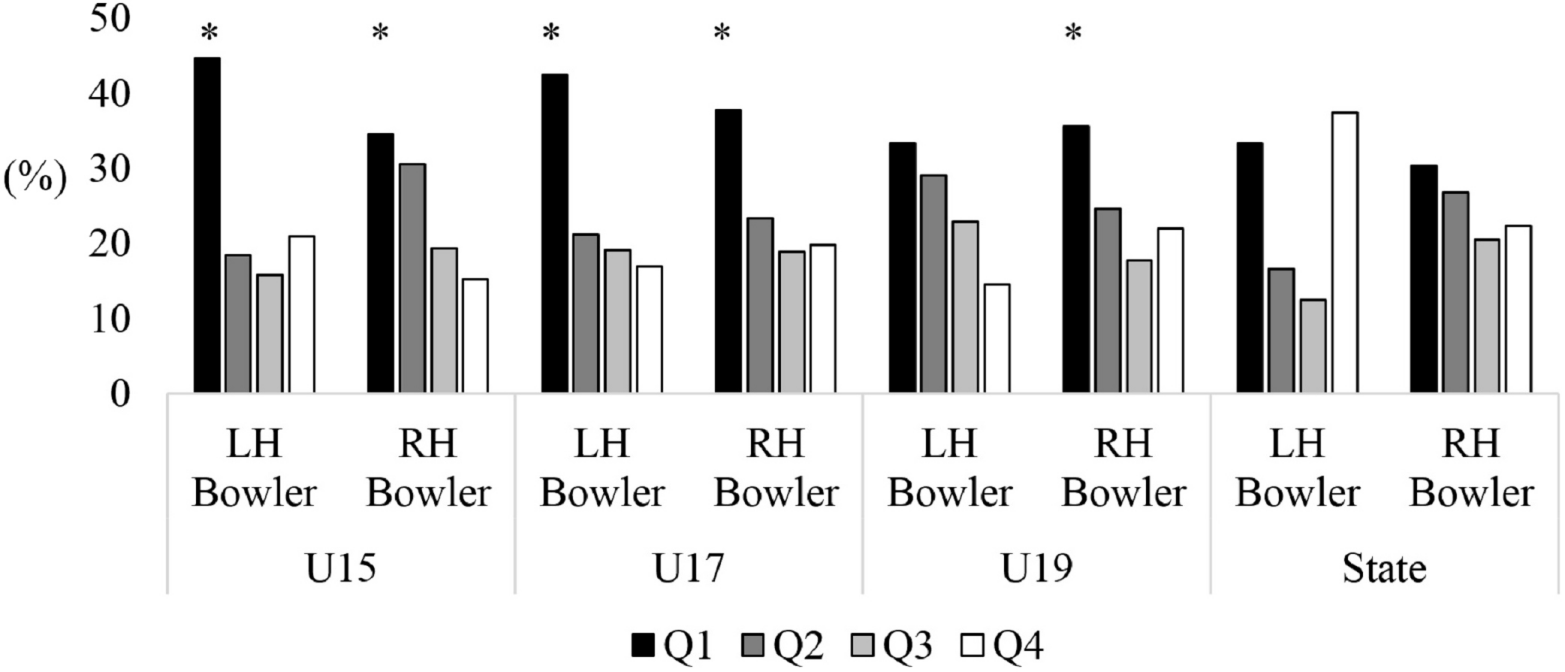
Comparison of birth quartiles and handedness for primary skilled pace bowlers, spin bowlers and all-rounders for junior representative male cricketers. *Statistically significant finding (*p* < 0.05).

Analysis of bowler’s handedness found significant effects for male left-handed (*X*^2^ = 8.12, *p* < 0.05 [Q1: 44.7%; Q2: 18.4%; Q3: 15.8%; Q4: 21.1%]) and right-handed (*X*^2^ = 12.45, *p* < 0.05 [Q1: 34.7%; Q2: 30.6%; Q3: 19.4%; Q4: 15.3%]) U15 bowlers, left-handed (*X*^2^ = 7.89, *p* < 0.05 [Q1: 42.6%; Q2: 21.3%; Q3: 19.1%; Q4: 17.0%]) and right-handed (*X*^2^ = 18.48, *p* < 0.05 [Q1: 37.8%; Q2: 23.4%; Q3: 18.9%; Q4: 19.9%]) U17 bowlers, and right-handed (*X*^2^ = 13.25, *p* < 0.05 [Q1: 35.6%; Q2: 24.6%; Q3: 17.8%; Q4: 22.0%]) U19 bowlers. In all groups, Q1 players were over-represented. No difference was found for left-handed (*X*^2^ = 3.83, *p* = 0.28 [Q1: 33.3%; Q2: 29.2%; Q3: 22.9%; Q4: 14.6%]) U19 level bowlers, or left (*X*^2^ = 4.33, *p* = 0.23 [Q1: 33.3%; Q2: 16.7%; Q3: 12.5%; Q4: 37.5%]) or right-hand *X*^2^ = 2.64, *p* = 0.45 [Q1: 30.4%; Q2: 26.8%; Q3: 20.5%; Q4: 22.3%]) State level bowlers (see Fig. 5).

**Figure 6:**
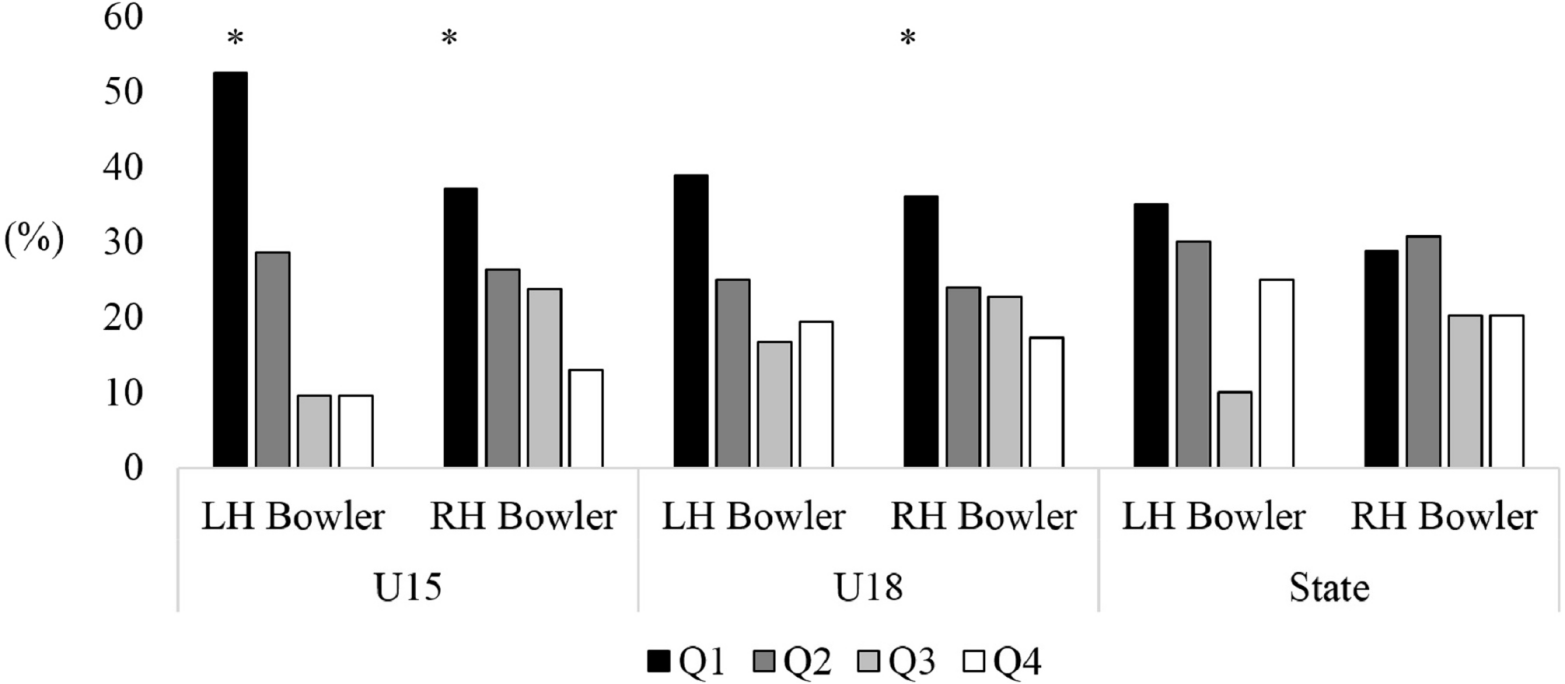
Comparison of birth quartiles and handedness for primary skilled pace bowlers, spin bowlers and all-rounders for junior representative female cricketers. *Statistically significant finding (*p* < 0.05).

For female bowlers, significant effects were found for left-handed (*X*^2^ = 10.43, *p* < 0.05 [Q1: 52.4%; Q2: 28.6%; Q3: 9.50%; Q4: 9.50%]) and right-handed (*X*^2^ = 30.67, *p* < 0.05 [Q1: 37.0%; Q2: 26.3%; Q3: 23.7%; Q4: 13.0%]) U15 bowlers, and right-handed (*X*^2^ = 19.27, *p* < 0.05 [Q1: 36.1%; Q2: 23.9%; Q3: 22.7%; Q4: 17.3%]) U18 bowlers. Similar to batters, all Q1 was the most represented in each of these groups, while Q4 was the least represented. No difference was reported for left-handed (*X*^2^ = 4.22, *p* = 0.24 [Q1: 38.9%; Q2: 25.0%; Q3: 16.7%; Q4: 19.4%]) U18 level bowlers or left (*X*^2^ = 2.80, *p* = 0.42 [Q1: 35.0%; Q2: 30%; Q3: 10.0%; Q4: 25.0%]) or right-handed (*X*^2^ = 3.92, *p* = 0.27 [Q1: 28.8%; Q2: 30.8%; Q3: 20.2%; Q4: 20.2%]) State level bowlers (see Fig. 6).

## Discussion

The aim of this study was to investigate whether there is a RAE in Australian cricket talent pathways. Secondly, what influences do moderating factors have on relative age on those who are selected? As predicted in the hypothesis, the main findings were (1) that a RAE was present for both male and female junior-state representative levels, across all age level competitions; (2) the RAE was less pronounced at a senior state level for both male and females; (3) for males, the effect was most prevalent for junior batters and pace bowlers (U15, U19; U15, U17 & U19 level respectively), and all-rounders (U15 and U17 level); (4) for females, all-rounders (U15 and U18) were most affected across both age groups, while batters (U15), spin bowlers (U15) and wicketkeepers (U18) were only impacted at one stage of the pathway; (5) left-handed batters and bowlers were not affected at the U19 level, unlike their right-handed counter-parts.

### Age and sex

Findings of RAE in male cricketers are consistent with the only other cricket study of this nature, which recognized a RAE in a UK County competition (Edwards, 1994). However, this is the first study to identify the prevalence among all major levels of the junior talent pathway for both male and female cricketers. Those born in the first quarter (Q1) of the playing year were significantly over-represented at the male U15 (36.1%), U17 (35.5%), and U19 level (34.2%), and female U15 (37.7%) and U18 level (36.9%). No differences were found for male (31.2%) or female (28.4%) cricketers playing at the senior state level. Interestingly, the magnitude of RAEs in cricket only gradually decrease as player’s reach the senior level. Two explanations are put forward to explain this gradual decline in RAE. Firstly, once players are identified as ‘talented’ and playing in the National junior competition, it is suggested that coaches are more likely to continue providing resources, access to higher level coaching, and more playing opportunities to these players. This can be further evidenced by a brief analysis of all U17 and U19 male players in this dataset, revealing that 61.6% played in more than one competition and/or year of the National Championships. Secondly, physical maturation, and the associated morphological and cognitive development, may play a role in technical execution and decision-making that underpin skilful performance (Schorer et al., 2009).

### Cricket-specific skills

All-rounders born in the first quartile of the sporting year were over-represented in every junior age group for both males (U15, U17 and U19 level) and females (U15 and U18 level). Additionally, male batters (except male U17) and pace bowlers were over-represented by relatively older players. It has been proposed that RAE may have a greater impact in sports with specific skills that rely on superior physical attributes (i.e., strength, speed, or power). Interceptive timing tasks, such as batting, would likely benefit from greater strength and power production (Miyaguchi & Demura, 2012), due to its role in producing faster bat swing velocity (DeRenne & Szymanski, 2009). In cricket, the ability to produce a high peak bat velocity alongside superior bat-ball contacts, are important factors when playing attacking strokes (Connor, Farrow & Renshaw, 2018). For example, Penn & Spratford (2012) highlighted in their review that state level batsmen exhibit lower peak bat velocity (15.22 ± 2.96 m/s; Elliott et al., 1993) than international level batsmen (21.20 ± 1.80 m/s; (Stuelcken, Portus & Mason, 2005) when playing an off-drive against bowlers. However, bat swing velocity is not the sole factor in successfully intercepting an object powerfully. It is imperative to note that successfully intercepting a cricket ball also requires superior visuomotor skills (Connor, Farrow & Renshaw, 2018). Therefore, the performance benefits associated with early physical maturation are only likely to only be a temporary performance advantage. Further research is required to investigate how much of an impact physical maturity (i.e., anthropometric and morphology) has on junior cricket batting skill.

While there is scarce literature on the anthropometric characteristics of male or female junior cricket batters, there has been a greater focus on fast bowling. Specifically, the anthropometric and physical characteristics have been shown to be predictors of fast bowling skill in junior cricketers (Pyne et al., 2006; Loram et al., 2005; Wormgoor, Harden & Mckinon, 2010). This likely explains the over-representation of relatively older pace bowlers being selected across all levels of the male pathway in the current study. Interestingly, this is in contrast to female pace bowlers who were not significantly over-represented in first quartile births (see Table 3). Stuelcken, Pyne & Sinclair (2007) noted that only male fast bowlers had physical characteristics that were proportionately large relative to their height. It is therefore proposed that female pace bowlers are less affected by the maturation hypothesis, and thus RAE.

**Table 3:** Positional breakdown of Under-15 and Under-18 junior state level female cricketers over 4 years and their birth quartile.

Birth Quartiles											
	*n*	Q1		Q2		Q3		Q4		*X*^2^	*p*-value
**All-rounder**							
State Under-15^*^	186	77	(41.4%)	45	(24.2%)	44	(23.7%)	20	(10.8%)	35.29	<0.05
State Under-18^*^	169	66	(39.1%)	41	(24.3%)	41	(24.3%)	21	(12.4%)	24.11	<0.05
State Level	61	18	(29.5%)	16	(26.2%)	14	(23.0%)	13	(21.3%)	0.98	0.81
**Batter**											
State Under-15^*^	66	27	(40.9%)	10	(15.2%)	12	(18.2%)	17	(25.8%)	10.49	<0.05
State Under-18	67	23	(34.3%)	14	(20.9%)	11	(16.4%)	19	(28.4%)	5.06	0.17
State Level	42	10	(23.8%)	9	(21.4%)	14	(33.3%)	9	(21.4%)	1.62	0.66
**Pace Bowler**											
State Under-15	76	28	(36.8%)	18	(23.7%)	17	(22.4%)	13	(17.1%)	6.42	0.09
State Under-18	102	34	(33.3%)	22	(21.6%)	18	(17.6%)	28	(27.5%)	5.76	0.12
State Level	52	16	(30.8%)	19	(36.5%)	9	(17.3%)	8	(15.4%)	6.62	0.09
**Spin Bowler**											
State Under-15^*^	27	6	(22.2%)	13	(48.1%)	5	(18.5%)	3	(11.1%)	8.41	<0.05
State Under-18	20	6	(30.0%)	7	(35.0%)	5	(25.0%)	2	(10.0%)	2.80	0.42
State Level	11	3	(27.3%)	3	(27.3%)	0	(0.0%)	5	(45.4%)	4.64	0.20
**Wicketkeeper**											
State Under-15	19	3	(15.8%)	6	(31.6%)	3	(15.8%)	7	(36.8%)	2.68	0.44
State Under-18^*^	21	11	(52.4%)	4	(19.0%)	5	(23.8%)	1	(4.8%)	10.05	<0.05
State Level	10	3	(27.3%)	3	(27.3%)	2	(0.0%)	2	(45.5%)	0.40	0.94

**Notes.**

* Statistically significant finding (*P* < 0.05).

Finally, female spin bowlers (U15 level; 48.1%) born in the second quartile of the year and wicketkeepers born in the first quartile of the year (U18 level; 52.4%) were significantly over-represented, although such findings were not reported for male spin bowlers and wicketkeepers. While studies examining the physical development of spin bowlers are limited, it is widely accepted that the ability to impart high revolutions on the ball, along with the angle and stability of the ball at the point of release, is associated with a skilful spin bowler (Spratford et al., 2015). Spratford and colleagues, in their examination of pathway (average age 19.6 ± 3.6 years) and ‘elite’ spinners (29.6 ± 7.8 years), found skill level differences in the ball velocity and angle of release between these two groups. It is currently unclear how influential anthropometric traits are to the development of this niche skill. Much like wicketkeeping, there may be a lack of competition pool to influence RAEs in this area. Within a cricket team, it is standard practice to only select one wicketkeeper and one spin bowler; in contrast to the 5–6 batters and 3–4 pace bowlers. Further research is required to explore the impact of physical maturation on spin bowler’s and wicketkeeper’s skill development in cricket.

### Handedness

Interestingly, there was no significant RAE for left-handers (batting or bowling) at the highest represented junior level for both male (U19) and females (U18), or at state level. This may suggest an advantage to left-handedness in cricket-specific skills as a player matures. There are two notable theories to explain the higher-than-expected prevalence of left-handers at high levels of competitions. The innate superiority hypothesis poses a neurophysiological explanation, such as a potentially better developed right hemisphere in left-handers, which might allow for superior performance in certain attentional tasks (Geschwind & Galaburda, 1987; Hagemann, 2009). However, another more commonly attributed explanation is the strategic advantage hypothesis, whereby unfamiliar movement patterns to the opposition (i.e., left-handers) may make it more difficult to perceive cues and anticipate opposition actions (Goulet, Bard & Fleury, 1989; Müller, Fadde & Harbaugh, 2017). Given the number of left-hand dominant batters and bowlers, opportunities to play against right-handers would be far more regular, while right-handers would have limited playing opportunities against left-handed opponents (Hagemann, 2009). In support of this argument was the findings from the 2003 Cricket World Cup, showing that left-handed batters were more successful in scoring runs than their right-handed counterparts (Brooks et al., 2004). Interestingly, the most successful teams were also reported to have close to 50% of the side being left-handed. Given there is a RAE for both left and right-handers during the junior levels of the national competition, the advantages of left-handedness may not be pronounced until players reach senior levels of competition. More research is warranted to examine the performances of different handedness in cricket competitions across different age levels.

## Conclusion

The results of this study suggest that RAE is most prevalent around the 15-year-old competition age group, for both male and female cricketers. This effect gradually declines in magnitude as player’s age and transition through to the FTEM cricket pathway (Gulbin et al., 2013). All-rounders, batters and pace bowlers, who are potentially advantaged by early physical maturity, demonstrated the greatest frequency of RAE across age groups. Given the nature of these cricket specific-skills, it is proposed that the maturation hypothesis explains why these skills (i.e., batting and pace bowling) are significantly impacted, while others less reliant on factors underpinned by physical prowess are not (i.e., spin bowling, wicketkeeping). Interestingly, once players reached the U19 age level competition, the RAE was not evident for left-handed batters or bowlers. This finding provides further evidence that the ‘fighting hypothesis’ circumvents the RAE bias in talent selection. It is also therefore critical that administrators, coaches and talent selectors are aware of the impact that RAE has on both male and female players as they traverse the talent pathway. Indeed, Mann & Van Ginneken (2017) demonstrated that talent selectors could successfully overcome RAE bias for athlete selection by providing junior players with a jersey number that corresponded to their age relative to their teammates. For example, in a group of 18, the oldest player would wear a jersey numbered 1, while the youngest players would wear a jersey numbered 8. This approach was effective in reducing the prevalence of RAE in talent selection by overtly emphasising relative age and providing additional context (i.e., individual age in relation to the selection cohort) to players performing skilfully. This highlights the need for coaches to be overtly aware of the relative age of players when conducting talent selection practices. It is also recommended that administrators take proactive steps to limiting the prevalence of RAE. For example, given the recent addition of an underage team into certain levels of the National championships competition (e.g., U16 National team competing in the U17 State Nationals competition), talent selectors should evaluate whether this has increased the selection of relatively younger players into State teams; based on the assumption that relatively older players are being selected into the National underage team.

## Supplemental Information

Dataset S1 Raw data of birth quartile, handedness, playing position and playing age group

### Additional Information and Declarations

#### Data Availability

The following information was supplied regarding data availability: The raw measurements are available in Dataset S1. The raw data shows the number of participants in born in each quartile. This data was used for statistical analysis to compare the distribution of birth quartiles across age groups.
